# Spina Bifida Defying Folic Acid Supplementation

**Published:** 2012-07-01

**Authors:** V Raveenthiran

**Affiliations:** SRM Medical College and Hospital SRM University, Chennai, India.

 (Athena stands for abbreviation of Abstracting and Thoughtful Evaluation of Neonatal Articles; but it is also personified by the contributor. Like Athena of Greek mythology, she distills wisdom from published literature)

Pre-conceptional supplementation of folic acid is well known to reduce the incidence of spina bifida. But Athena is frequently perplexed to see this anomaly occurring despite folate supplementation. In a largest Canadian study, De Wal et al [1] screened 1.9 million live births to study the effect of mass fortification. On comparing pre and post fortification periods they noted only 46% reduction in the incidence of neural tube defects (NTD). What happens to the remaining 54%? Why do they defy “folic acid” logics? Recently several publications have shed more light on this intriguing question.

Researchers at the University of Tokyo [2] studied serum level of folic acid in 254 pregnant women and correlated it with dietary habits and total folate intake per day. They found that serum folate was significantly lower in women taking green tea and oolong tea than those taking black tea. This effect was noted even at 100 to 130 ml of tea intake per day. Reduced bioavailability of folate in these women has been attributed to interaction of tea catechin (tannin) and dietary folate. Epigallo Catechin Gallate (EGCG), a tea catechin, is found to inhibit Dihydroxy Folate Reductase. This enzyme is essential to convert dietary folic acid into Tetrahydroxy Folate which is the active form. Even if a pregnant woman takes adequate folate supplementation, concurrent ingestion of tea will reduce its bioavailability and results in deficiency state. Another team of Boston epidemiologists [3] independently verified these findings from clinical point of view. In that study, they compared mothers of 518 spina bifida cases with 6424 controls for tea consumption. Among women with folic acid intake greater than 400 µg per day, consumption of 3 or more cups of tea per day significantly increased the risk of fetal spina bifida. Athena considers it prudent to advise newlywed women to avoid tea. In India, tea is popular in some northern states while coffee is preferred in southern states. A comparison of the incidence of NTD in these states would probably be an interesting study.

Emejulu and co-workers [4] from south-east Nigeria were intrigued by the high incidence of spina bifida in areas endemic of malaria. What could the relationship between plasmodium and neural tube? Further inquiry suggested that antimalarial drugs rather than plasmodium could be the culprit. All the 41 mothers who had anomalous children had taken antimalarial drugs during first trimester. Chloroquine and other antimalarial drugs act by blocking folate metabolism of plasmodium. Analogically they can also block folate metabolism of pregnant women. Thus concurrent administration of antimalarial drugs reduces the bio-utilization of folates thereby increase the risk of malformations. Athena considers this as an important discovery with direct implication to South-east Asian and African countries. In the background of this new study she questions the prudence of Mass Drug Administration under National Malaria Eradication Program (NMEP).

A recent study from Texas [5] indicates that spina bifida can occur despite folate supplementation if target cells are deprived of the vitamin. These researchers have identified 3 types of folate receptors (FOLR1, FOLR2, FOLR3) in cell membranes. These receptors are essential to maintain critical levels of intracellular folate by unidirectional transport and cytoplasmic release of folic acid. Embryos carrying defective genes codifying these receptor proteins are more likely to develop meningomyelocele despite maternal supplementation of folates. Texas researchers tested this hypothesis in 329 affected child-parent trios, 281 affected child-parent duos and 92 controls. They identified several alleles of the receptor genes that are associated with increased risk of meningomyelocele. Athena is curiously interested to know how the findings of this basic research can be translated into clinical use.

Folic acid prevents NTD by taking part in the repair of damaged DNA of neural crest cells. It is then logical that a newborn may develop spina bifida despite maternal folate supplements if the collateral damage of DNA exceeds the reparative process. Oxidative stress and free radicals produced thereof are well known to cause DNA damage. Organic solvents which are commonly used in inks, pesticides, fuels, cosmetics, and dyes are known to cause oxidative stress. Therefore, the US National Birth Defects Prevention Study Group [6] investigated the relationship between NTD and organic solvents. The analysis included 511 mothers of NTD cases and 2972 controls. Women were enrolled if they had been occupationally exposed to organic solvents for at least 30 days from 1 month preceding the estimated date of conception through the end of first trimester. Nearly 14% of affected mothers had been exposed to organic solvents as against only 8% of control group. Among the various types of solvents, Chlorinated chemicals such as carbon tetrachloride, chloroform and trichloroethylene carried a higher risk of NTD than aromatic solvents such as Benzene, Xylene and Toluene. Interestingly, 75.6% of affected mothers had received preconception folate supplements!

Analogous to organic solvents hydrocarbon exhaust of automobiles can also cause oxidative stress. Diesel exhaust particles (DEP) are frequently less than 0.1 micron in size. Owing to this extremely small size they enter circulation if inhaled and easily cross blood-brain barrier and placental barrier. Simsek et al [7] have recently studied the teratogenic effect of DEP in chick embryo model. They injected DEP of 10, 50, 100 and 200 µm sizes into 30-hour-old chicks. Examination of the embryos at 72 hours of incubation revealed that the group exposed to DEP was at high risk of malformations. NTD was noted in 18% of embryos exposed to DEP while in none of the control group. The risk of malformation was higher with increasing particle size. About 40% of embryos developed NTD when exposed to 200 µm DEP while the incidence was as low as 8 to 13% for smaller size particles. It is to be seen if this DEP induced NTD can be prevented by folate supplements. Athena considers this as a seminal work in view of ubiquitous air pollution of the modern world. She is interested to know if the incidence of NTD differs between rural and urban populations as the latter is more exposed to DEP. The common denominator of teratogenicity of organic solvents or hydrocarbons is the DNA damage induced by oxidative stress and free radicals. Neural crest cells are more vulnerable for oxidative stress not only because of their high metabolic demand but also because of paucity of endogenous scavengers such as Vitamin C. Athena wonders if we should supplement Vitamin C along with folates to prevent DNA damage that bypasses folate mechanism.

Athena has seen high incidence of NTD following threatened abortions and assisted reproduction techniques such as in-vitro fertilization. It is attributed to high doses of progesterone used to avoid fetal loss in these clinical scenarios. Iqbal et al [8] studied the effect of high dose progesterone on the development of neural tube using chick embryo model. They found NTD in 75% of the eggs treated with high dose progesterone while none of control group embryos developed this malformation. When they supplemented 5 µg of folic acid the incidence of NTD dropped to 58%. It appears that both progesterone and folic acid mutually blocking each other by competing for the same receptor. The common receptor is identified as GABA-A (Gamma Amino Butyric Acid receptor A). GABA is well known to be an important neurochemical. GABA-A agonists are known to increase the risk of NTD. Progesterone acts as GABA-A agonist while folic acid acts as antagonist at the receptor. By competitive binding progesterone also reduces the placental uptake and efflux of folic acid. Thus, high levels of maternal progesterone may produce fetal deficiency of folates despite supplementations. Currently 400 µg per day is the recommended dose of folic acid supplementation. It remains to be seen if increasing the dosage of folate supplement would oppose the teratogenic effect of progesterone by competitive receptor blocking.

Athena now realizes that there are many a slip between cup and the lip. Taking folate supplements is not sufficient to prevent NTD. The process requires understanding of a complex sequence involving adequate absorption, optimal bioavailability, drug interactions, presence of appropriate receptors, competitive receptor blocking and degree of collateral damage to DNA.

## Footnotes

**Source of Support:** Nil

**Conflict of Interest:** None declared

